# Evaluation of Chlamydia trachomatis screening from the perspective of health economics: a systematic review

**DOI:** 10.3389/fpubh.2023.1212890

**Published:** 2023-10-10

**Authors:** Huan Yao, Cuizhi Li, Fenglin Tian, Xiaohan Liu, Shangfeng Yang, Qin Xiao, Yuqing Jin, Shujie Huang, Peizhen Zhao, Wenjun Ma, Tao Liu, Xiaomei Dong, Cheng Wang

**Affiliations:** ^1^Department of Public Health and Preventive Medicine, School of Medicine, Jinan University, Guangzhou, China; ^2^Dermatology Hospital, Southern Medical University, Guangzhou, China

**Keywords:** Chlamydia trachomatis, mass screening, cost-effectiveness analysis, cost-benefit analysis, cost-utility analysis, sexually active people, pregnant women

## Abstract

**Background:**

Most Chlamydia trachomatis (CT) infections are asymptomatic. The infection can persist and lead to severe sequelae. Therefore, screening for CT can primarily prevent serious sequelae.

**Aim:**

To systematically evaluate CT screening from the perspective of health economics, summarize previous findings from different target populations, and make practical recommendations for developing local CT screening strategies.

**Methods:**

PubMed, Web of Science, Embase, Cochran Library, and National Health Service Economic Evaluation Database (Ovid) were searched from January 1, 2000, to March 4, 2023. Studies reporting the cost-effectiveness, cost-benefit, or cost-utility of CT screening were eligible to be included. A narrative synthesis was used to analyze and report the results following the PRISMA guidelines. The Consensus on Health Economic Criteria (CHEC) list was used to assess the methodological quality of included studies.

**Results:**

Our review finally comprised 39 studies addressing four populations: general sexually active people (*n* = 25), pregnant women (*n* = 4), women attending STD and abortion clinics (*n* = 4), and other high-risk individuals (*n* = 6). The total number of participants was ~7,991,198. The majority of studies assessed the cost-effectiveness or cost-utility of the screening method. The results showed that the following screening strategies may be cost-effective or cost-saving under certain conditions: performing CT screening in young people aged 15–24 in the general population, military recruits, and high school students; incorporating CT screening into routine antenatal care for pregnant women aged 15–30; opportunistic CT screening for women attending STD and abortion clinics; home-obtained sampling for CT screening using urine specimens or vaginal swab; performing CT screening for 14–30-year-old people who enter correctional institutions (i.e., jail, detention) as soon as possible; providing CT screening for female sex workers (FSWs) based on local incidence and prevalence; adding routine CT screening to HIV treatment using rectal samples from men who have sex with men (MSM).

**Conclusion:**

We found that CT screening in general sexually active people aged 15–24, military recruits, high school students, pregnant women aged 15–30, women attending STD and abortion clinics, people entering jail, detention, FSWs, and MSM has health economic value. Due to the different prevalence of CT, diversities of economic conditions, and varying screening costs among different populations and different countries, regions, or settings, no uniform and standard screening strategies are currently available. Therefore, each country should consider its local condition and the results of health economic evaluations of CT screening programs in that country to develop appropriate CT screening strategies.

## 1. Introduction

Chlamydia trachomatis (CT) infection is one of the four most common curable sexually transmitted infections (STIs) (trichomoniasis, chlamydia, gonorrhea, and syphilis) worldwide (1). The global burden of CT remains high (2). Approximately more than 100 million new cases of CT are detected annually. In 2020, ~128 million new cases of CT were reported by World Health Organization (WHO), far more than syphilis cases (82 million) and gonorrhea cases (7 million) (3). Based on the surveillance data of sexually transmitted diseases (STDs) from 2009 to 2016, the average global prevalence of CT was 3.8% in 15–49-year-old women and 2.7% in men (2). The prevalence of Chlamydia infection varies worldwide, with the lowest in South-East Asia (1.5% in women and 1.2% in men) and the highest in American women (7.0%) and African men (4.0%) (4). The economic burden is high according to the estimated cost of CT infection in some countries. The direct lifetime medical cost of CT infection was about 516.7 million USD from 2002 to 2011 in the United States, and it was the most costly non-viral STI (5). In Canada, the total estimated cost of CT infection in people aged 10 to 39 years was over $1.0 billion, or $56.4 million per year from 1991 to 2008 (6).

Chlamydia can be transmitted during vaginal, anal, or oral sex. About 75% of infected women and nearly 50% of infected men have no symptoms (7). However, the infection can persist and lead to severe sequelae. In women, untreated CT infections can progress to pelvic inflammatory disease (PID), leading to chronic pelvic pain (CPP), infertility, or ectopic pregnancy (EP) (8). The newborns can be infected through vertical transmission, causing neonatal conjunctivitis or pneumonia (9, 10). In men, CT infections can cause non-gonococcal urethritis or epididymitis (11). Moreover, CT infection can facilitate HIV transmission and may potentiate the risk of cervical cancer (12, 13). CT infection seriously affects people's health and lives and has become a significant public health problem worldwide. Furthermore, occult symptoms increase the likelihood of transmission. Therefore, early screening to identify cases of CT infection and timely treatment are critical measures to control disease transmission and reduce sequelae.

Currently, many developed countries (i.e., North America, Europe, and Oceania) have explored the strategies of CT screening, but few countries in Asia, such as China, have done the same. Some countries have even published CT screening guidelines (14–17). Herein, both the United States and Australia recommended screening different target populations, such as pregnant women, MSM, and HIV-infected individuals. However, most of these guidelines were developed according to national characteristics and might not be universally applicable. In addition, the specific effects of CT screening strategies on reducing infections and health resource consumption in different countries remain unknown. Therefore, systematic health economic evaluation is essential to comprehensively evaluate the effectiveness of different screening strategies, optimize screening strategies, and ensure rational allocation of limited health resources. Currently, there is only one systematic review related to the health-economic evaluation of CT screening (18). Nevertheless, it did not include the latest original studies published after 2004, and it included non-screening methods like diagnostic and therapeutic methods. Considering the problems mentioned above, a recent and focused systematic review is needed to measure the efficacy of current CT screening methods from an economic evaluation viewpoint. Our research questions were: (1) Is screening cost-effective as an intervention for probable CT infection? (2) Have different screening programs different cost-effectiveness (benefit and utility)? (3) Is CT screening beneficial from the standpoint of health economics to guide health decision-making?

We aimed to summarize the results of health economic evaluation of CT screening in different populations worldwide and provide a reference to develop scientific and appropriate local CT screening strategies.

## 2. Methods

This systematic review followed the Preferred Reporting Items for Systematic Reviews and Meta-Analyses (PRISMA) guidelines. The PICOS criteria (participants/patients, intervention, comparison, outcomes, and study design) were used to guide the search strategy. Database search, study selection, data extraction, and quality assessment were conducted independently by two investigators (YH and LZ). Disagreements were resolved through discussions and consultation with a senior investigator (DM).

### 2.1. Search strategy and study selection

A literature search was conducted through five electronic databases, including PubMed, Web of Science, Embase, Cochrane Library, and the National Health Service Economic Evaluation Database. The search results were limited to January 1, 2000, to March 4, 2023.

The PICOS-style search terms were used comprising three main areas: P (Chlamydia trachomatis), I (screening), and S (cost-effectiveness, cost-utility, or cost-benefit). Comprehensive search strategies for each database are provided in Supplementary material. The reference lists of all included articles and relevant reviews were also reviewed for any other papers that might not have been identified in the databases searches.

Retrieved articles were imported into the Endnote X9.1 reference management system, and duplicates were removed. Study selection was initially performed based on titles and abstracts. Then, the full texts of selected articles were studied to determine eligibility.

### 2.2. Selection criteria

The inclusion criteria were as follows: (1) original studies that performed a cost-effectiveness analysis (CBA), a cost-benefit analysis (CEA), or a cost-utility analysis (CUA) on CT screening; (2) data collection and analysis were based on either an economic model or a trial; (3) the study was published between January 1, 2000 and March 4, 2023; and (4) the full text was accessible and written in English.

The following studies were excluded: (1) studies reporting the evaluations of cost or effectiveness separately; (2) studies focusing on economic evaluation of STD screening or joint screening (i.e., gonorrhea and chlamydia screening), without separately reporting the results of CT screening; or (3) reviews, commentaries, editorial, letters, or reports.

### 2.3. Data extraction and synthesis

Study information, including authors' names, year of publication, population, age range, sample size, type of economic evaluation, time horizon and discount rate, outcome measures, and main CE results, were extracted into a predefined form. A narrative synthesis was used due to the diversity of studies.

### 2.4. Quality assessment

The Consensus Health Economic Criteria (CHEC) list was applied to assess the methodological quality of economic evaluations. This CHEC instrument comprises a 19-item list (19) on study design (4 items), time horizon, actual perspective, cost evaluation (5 items), outcome measurements (3 items), discounting, conclusion, generalization, conflict of interest, and ethical issues. As the CHEC list does not specify summary scores, score limits were defined by investigators. Each item was rated with three possible answers: N = no, with no points; U = unclear, with half a point; and Y = yes, with one point. The total score <10, 10–14.5, and > 14.5 indicated low, moderate, and high-quality economic evaluation, respectively (20). The findings of the quality assessment did not determine the inclusion or exclusion of studies.

## 3. Results

### 3.1. Study selection

**Figure 1:**
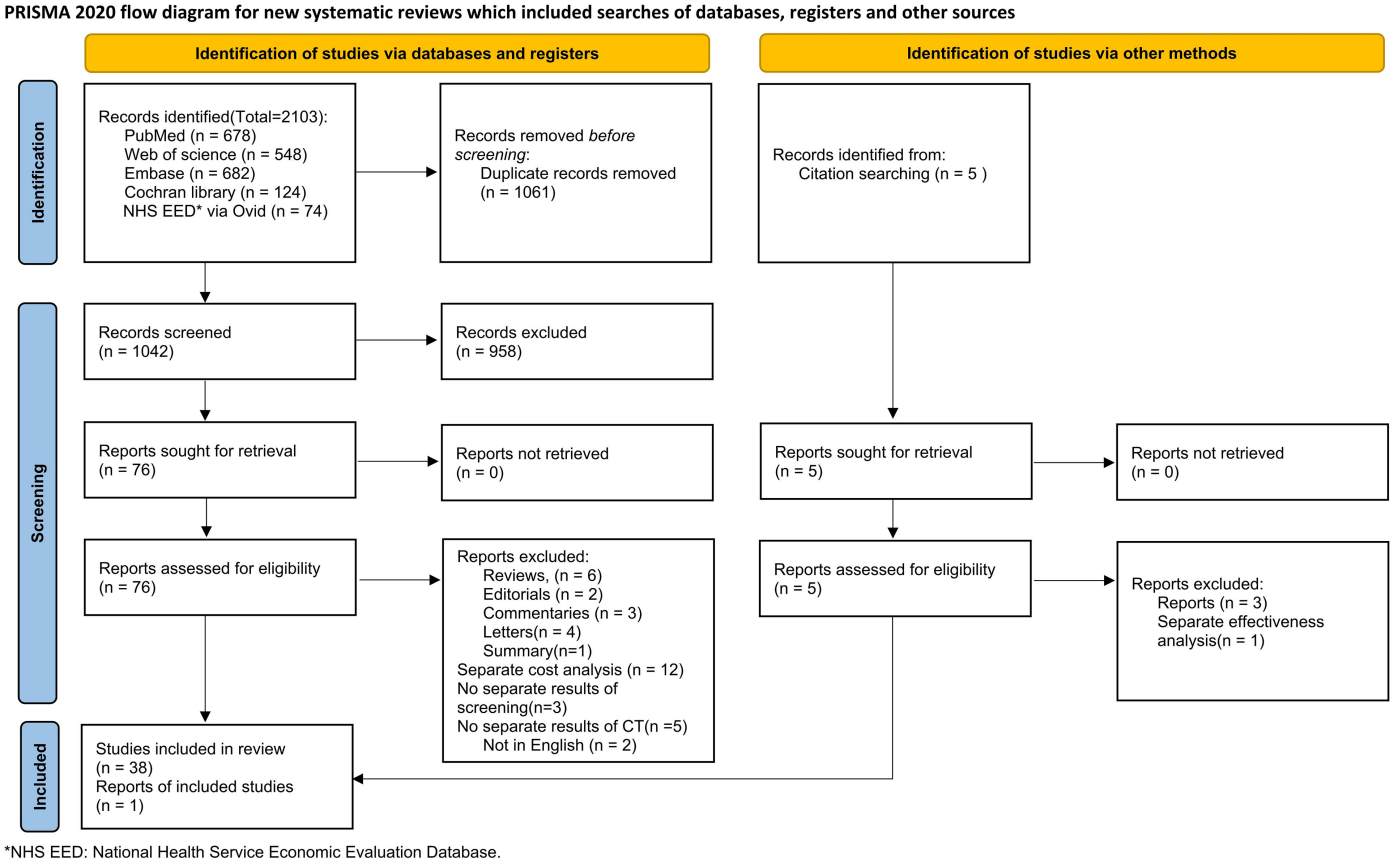
PRISMA flow diagram of study selection.

The PRISMA diagram shows the details of the systematic review (see Figure 1). In total, 2103 records were retrieved from databases. After removing 1,061 duplicates, 1,042 records were screened based on titles and abstracts, 76 were eligible for full-text review, and 38 articles were included. One additional record was identified by searching the reference lists. Finally, we included 39 studies in the quality assessment and narrative synthesis.

### 3.2. General characteristics of the included studies

A summary of included studies is presented in Table 1, and their main characteristics are provided in Table 2. Most studies (35 out of 39) were from North America and Europe. Most studies were from the USA (15) and the Netherlands (9). Only one study was from China.

**Table 1:** Summary of included studies.

**Population**	**Countries**	**Number of studies**	Total sample size^*^	**Study design**	**Data sources for costs and comes**	**Perspective**
Sexually active people	9 USA, 2 UK, 7 Netherlands, 2 Canada, 1 Hungary, 1 Sweden, 1 Denmark, 1 Australia, 1 Ireland	25	542, 840	10 Dynamic model, 14 Static model, 1 Trial	2 Primary, 14 Secondary, 9 (Primary and Secondary)	11 Healthcare, 10 Societal, 1 Government, 1 Military and civilian, 2 not reported
Pregnant women	1 Hungary, 1 Australia, 1 Netherlands, 1USA	4	7, 240, 741	4 Static model	4 (Primary and Secondary)	4 Healthcare
Women attending STD and abortion clinics	1 UK, 1 China, 1 USA, 1 Canada	4	75, 770	1 Dynamic model, 3 Static model	1 Secondary, 3 (Primary and Secondary)	2 Healthcare, 1 Societal, 1 Correctional institution and healthcare system
Other high-risk individuals	4 USA, 1 Australia, 1 Netherlands	6	131, 847	3 Dynamic model, 3 Static model	2 Secondary, 4 (Primary and Secondary)	1 Healthcare, 3 Societal, 2 not reported

^*^An approximate estimate value.

**Table 2:** Main characteristics of included studies.

**Author**	**Year of publication**	**Population**	**Age range, years**	**Sample size (sex ratio)**	**Type of economic evaluation**	**Currency (year)**	**Time horizon and discount rate**	**Sensitivity analysis (✓= done, × = not done)**	**Outcome measures**	**Evaluation indicators**	**Main CE results**	Screening was found to be cost-effective (✓= yes, ✓/ × ^*^, × = no)
**Sexually active people**											
Howell et al. (21)	2000	Army female recruits	≤ 25	>18,000	CEA	USD (1999)	5 years; 3% costs	✓	MOA	ICER	Military perspective: cost-saving for female recruits aged ≤ 25; Civilian perspective: cost-saving for all female recruits.	✓
Postma et al. (22)	2000	Asymptomatic female	15–34	100	CEA	USD (1999)	NS; 3% costs	✓	MOA	CER	Cost-saving for female aged 15–19 (at ligase chain reaction test costs of US$ 17); cost- saving for female aged 15–29 (the test cost is lower and the sensitivity is raised).	✓/ ×
Welte et al. (23)	2000	Sexually active male and female	15–24	10,000 (1:1)	CEA	USD (1997)	42 years 3% costs and 3% effects	✓	MOA	CER	US$492 or US$1,086 (cost-saving)/ MOA during the first 10 years.	✓
Goeree et al. (24)	2001	Young female	15–24	NS	CEA	CAD (1999)	10 years; NS	✓	MOA	ICER	ICER = $1,873/CT case averted for high-risk symptomatic female (swab-based screening and treatment vs. swab-based diagnostic testing).	✓
Nyári et al. (25)	2001	Asymptomatic female	<20	1, 300	CEA	USD (NS)	NS; NS	✓	CT cases prevented	ICER CER	Cost-effective for female age <20 if the infection, PID, tubal infertility rate exceeded 16.7%, 24%, 25%, respectively (Amplified Gen-Probe assays with PN vs. ELISA method).	✓/ ×
Van Valkengoed et al. (26)	2001	Asymptomatic female	15–40	5, 541	CEA	USD (1999)	NS; 3% costs	✓	QALY	CER	Net cost $15,800/MOA for female aged 15–40 at a prevalence of 2.9%; cost-saving at a rate of 41.8%.	✓/ ×
Wang et al. (27)	2002	High school adolescents (male and female)	NS	Female:1,402 Male: 1,251	CEA	USD (1997)	NS; NS	✓	MOA	ICER	ICER = $1,524/PID averted (school-based vs. non-school based).	✓
Ginocchio et al. (28)	2003	Asymptomatic young male	NS	100,000	CEA	USD (2000)	PID (1 year); infertility (10 years) EP and CPP (5 years) 3% costs and 3% outcomes	✓	MOA	ICER	Cost-effective for LE-LCR (prevalence: 5%); cost-effective for LCR (LCR cost ≤ $18 or prevalence: 49%).	✓/ ×
Hu et al. (29)	2004	Sexually active female	15–29	100,000	CEA/CUA	USD (2000)	Lifetime; discounting applied, rates NS.	✓	QALY MOA	ICER ICUR	ICUR = $7,490 (<threshold $50000)/QALY for female aged 15–29 (semiannual screening for those with a history of infection).	✓
Novak et al. (30)	2004	Asymptomatic young male and female	20–24	200 (1:1)	CEA	USD (2001)	NS; NS	✓	MOA	ICER	Cost -effective for asymptomatic young population (prevalence: female>5.1%, male>12.3%; participation rate: 55%).	✓/ ×
Van Bergen et al. (31)	2004	High risk young female	15–29	446	CEA	EUR (2001)	2 years; 4% costs and 4% effects	×	MOA	CER	Pharmacy-based screening using mailed samples: cost-saving for female aged 15–29 at a high PID-risks (40%); cost-saving for female aged 15–24 at PID-risk (20%) (lower laboratory evaluation costs).	✓/ ×
Andersen et al. (32)	2006	Young male and female	15–24	10, 000 (1:1)	CEA	USD (2002)	10 years; 3% costs and 3% effects	✓	MOA	ICER	ICER = $3186/MOA (home-sampling vs. in-office) in the first year; cost-saving to society in the fourth year.	✓
de Vries et al. (33)	2006	Young male and female	15–29	21,000	CEA	EUR (2002)	10 years; 4% costs and 4% effects	✓	MOA	CER	Net cost €373 (€274)/MOA (PID) averted for male and female aged 15–29; partner treatment and reminder could improve the cost-effectiveness.	×
Walleser et al. (34)	2006	Young female consulting a general practitioner	≤ 25	NS	CUA	AUD (NS)	25 years; 5% costs and 5% effects	✓	QALY	ICUR	ICUR = AU$2,968/QALY for female aged ≤ 25 (annual opportunistic screening vs. no screening).	✓
Adams et al. (35)	2007	Heterosexual male and female	16–44	40, 000 (1:1)	CEA/CUA	GBP (2004)	10 years; 3.5% costs and 3.5% effects	✓	QALY MOA	ICER CER ICUR CUR	Annual screening: ICUR <accepted threshold £20, 000-£30, 000 for male and female aged <20 (PID regression ≥10%).	✓/ ×
Roberts et al. (36)	2007	Young male and female	16–24	50, 000	CEA	GBP (2005)	8 years; 3.5% costs and 3.5% effects	✓	MOA	ICER	ICER = £28, 900 (£22,300)/MOA for female and male (only female) (proactive screening vs. no organized screening).	×
Blake et al. (37)	2008	Female and male job training students	16–24	4, 000 (1:1)	CEA	USD (2005)	10 years; 3% costs	✓	MOA	ICER	Cost-effective and cost-saving for male individually and in combination (universal ECS NAAT, universal urine NAAT).	✓
De Vries et al. (38)	2008	Young male and female	15–29	100, 000 (1:1)	CUA	EUR (2002)	20 years; 4% costs and 4% effects	×	QALY	ICUR	ICUR <informal threshold €20,000/QALY (screening every 2 years).	✓
Nevin et al. (39)	2008	Male military recruits	≤ 24	NS	CEA	USD (2005)	10 years; 3% costs	✓	MOA	ICER	Cost-effective for male recruits: selective (ICER = $3,700/PID averted) and universal (ICER = $8,200/PID averted) screening with female PN.	✓
Huang et al. (40)	2011	Sexually active young female	NS	10, 000	CEA	USD (2010)	NS; 3% costs and 3% effects.	✓	MOA	ICER	ICER = $1,155/PID averted (internet-based, self- swab screening vs. traditional, clinic-based screening).	✓
Gillespie et al. (41)	2012	Young male and female	18–29	40, 000 (1:1)	CEA/CUA	EUR (2008)	10 years; 3.5% costs and 3.5% effects	✓	QALY MOA	ICER ICUR	ICER = € 94,717 (>threshold € 45,000)/ QALY (opportunistic screening vs. no screening).	×
Tuite et al. (6)	2012	Risk age groups of male and female with relatively high CT incidence	10–39	NS	CUA	CAD (1999)	10 years; 3% costs and 3% effects	✓	QALY	ICUR	ICUR = $2,910 (<Canada's per capita GDP/ QALY of $39,400) for asymptomatic male and female (enhanced screening vs. no change of screening).	✓
de Wit et al. (42)	2015	Young male and female	16–29	NS	CEA/CUA	EUR (NS)	10 years; 4% costs and 1.5% effects	✓	QALY MOA	ICER ICUR	Cost-effective only at a high levels willingness to pay for > €50,000/QALY and at a high participation (repeated round of screening vs.no screening).	✓/ ×
Wang et al. (43)	2021	High school students (male and female)	NS	NS	CUA	USD (2016)	20 years; 3% costs and 3% effects	✓	QALY	CUR	Cost-effective if the participation rate was improved from 3% to 7%; cost/QALY <$50,000 (participation rate >7%).	✓/ ×
Stoecker et al. (44)	2022	Black, sexually active male and female	15–24	Female: 13,419 Male: 16,181	CUA	USD (2018)	1 year; 3% effects	✓	QALY	CUR	CUR = $5,468 (<a standard threshold $50000)/QALY (the check it program).	✓
**Pregnant women**											
Nyári et al. (45)	2003	Pregnant women	15–24	790, 000	CEA	USD (NS)	1.5 years; NS	✓	CT cases prevented	ICER	Cost-effective if the diagnostic test cost ≤ US$10, infection rate>8.3% or the the probability of tubal infertility>20% (screening vs. neither testing nor treating).	✓/ ×
Ong et al. (46)	2015	Pregnant women	16–25	NS	CUA	AUD (2014)	1year; NS	✓	QALY	ICUR	Cost-effective for pregnant women aged 16–25 during one antenatal visit if prevalence >5% (screening all vs. selectively screening).	✓/ ×
Rours et al. (47)	2015	Pregnant women	NS	4, 055	CUA	EUR (NS)	NS; 4% partial costs and 1.5% effects	✓	QALY MOA	CUR	Net cost-saving for all pregnant women (test price <€20); more cost-saving for women aged <30 or with first pregnancies (sntenatal CT screening).	✓/ ×
Ditkowsky et al. (48)	2017	Pregnant women	15–24	6, 446, 686	CBA	USD (2015)	1 year; NS	✓	CT cases treated	Net cost-saving	Net cost-saving when prevalence>16.9%, WTP = 0 (prenatal screening vs. no screening).	✓/ ×
**Women attending clinics**											
Norman et al. (49)	2004	Women attending abortion, family planning clinics, etc.	Reproductive age	3,750	CEA	GBP (2001)	NS; 5% costs and 3% effects	✓	MOA	ICER	ICER = £258 (£433)/ MOA for women under 20 years (all patients attending abortion clinics).	✓
Chen et al. (50)	2007	Women seeking induced abortions	NS	2,020	CEA	RMB (2002)	NS; 3% costs	✓	MOA	ICER	Cost-saving 18,239 RMB/ PID averted (azithromycin-based prophylaxis vs. universal screening).	✓
Blake et al. (51)	2008	Women attending STD clinics	NS	10, 000	CEA	USD (2006)	10 years; 3% costs	✓	MOA	ICER	Cost-saving $2,384/PID averted (self -obtained vaginal AC2 strategy).	✓
Thanh et al. (52)	2017	Women attending STI clinics	NS	60,000	CUA	CAD (2016)	10 years 3% costs and 3% effects	✓	QALY	ICUR	Cost-effective if the CA$50,000 <WTP CA$100,000 (ICUR = CA$34,000, CA$49,000/QALY) (UG+SR, UG+UR vs. UG-only).	✓/ ×
**Other high-risk individuals**											
Blake et al. (53)	2004	Adolescent males	14–18	594	CEA	USD (2002)	10 years; 3% costs	✓	MOA	ICER	Cost saving $24,000 with 37 more PID and 3 more epididymitis averted (universal NAAT screening vs. selective IE screening).	✓
Kraut-Becher et al. (54)	2004	Young male and female	NS	20, 000 (1:1)	CEA	USD (2002)	NS; 3% costs	✓	MOA	ICER	Female: cost-saving if CT rate>7.7%, NG rate <3.7% (separate CT screening); Male: cost-saving if CT rate <3.3%; NG rate <3.8% (presumptive treatment).	✓/ ×
Gift et al. (55)	2006	Young male	Average 30	1, 000	CEA	USD (2001)	4 months; 3% costs	✓	MOA	ICER	Age-based screening of male <30 years old would have identified slightly fewer cases (47.7 vs. 49.9) at half cost ($13650 vs. $27060) of universal screening.	✓
Gift et al. (56)	2008	Young male and female	15–24	100, 000 (1:1)	CEA/CUA	USD (2006)	5 years; 3% costs and 3% effects	✓	QALY MOA	ICER ICUR	Screening male could be a cost-effective alternative to screening female when the prevalence in screened male was 86% higher than that of screened female.	✓/ ×
Wilson et al. (57)	2010	Female sex workers	NS	2, 000	CUA/CBA	AUD (NS)	NS; 3% costs and 3% effects	×	QALY	NMB CUR	ICUR = AU$600,000 (>assumed willingness to pay AU$5,000)/QALY; NMB = - AU$1.5 million/year (mandatory screening of FSWs at every 4 weeks).	×
Vriend et al. (58)	2013	MSM in care at HIV treatment centers	NS	8, 253	CUA	EUR (NS)	20 years; 4% costs and 1.5% effects	✓	QALY	ICUR	Adding routine screening was cost-saving if these patients seek little (30%) or no (0%) non-routine screening elsewhere.	✓/ ×

^*^✓/ × = to some extent completed (under certain assumptions and conditions, the intervention was found to be cost-effective). NS, not stated; CEA, cost-effectiveness analysis; CBA, cost-benefit analysis; CUA, cost-utility analysis; QALY, quality adjusted life year; MOA, major outcome averted; CER, cost-effectiveness ratio; ICER, incremental cost-effectiveness ratio; NMB, net monetary benefit; WTP, willingness-to-pay; PN, partner notification; PID, pelvic inflammatory disease; CPP, chronic pelvic pain; EP, ectopic pregnancy; ECS, endocervical; Teen HC, Teen Health Centers; ELISA, enzyme-linked immunosorbent assay; DIF, direct immunofluorescence assay; PCR, polymerase chain reaction; NAAT, nucleic acid amplification test; LE, leukocyte esterase; LCR, ligase chain reaction; LE-LCR, testing with a LE strip test of urine, followed by confirmatory LCR; In-office screening, an opportunistic screening in doctors' office; The Check It program, a bundled seek; test; and treat chlamydia prevention program for young Black men; MSM, men who have sex with men; AC2, the Aptima Combo 2 test is among a new generation of tests that use nucleic acid amplification and is FDA-cleared for use on endocervical; urine; and vaginal specimens; STI, Sexually transmitted infection; UG, universal urogenital; UR, universal rectal; SR, selected (exposure-based) rectal; USD, United State Dollar; EUR, Euro; GBP, pound sterling (Great Britain Pound); AUD, Australian dollars; CAD, Canadian dollars.

Twenty studies used both primary and secondary data, and 17 studies only used secondary data. The predominant type of economic evaluation was CEA (23 studies), followed by CUA (9 studies). Five studies performed both CEA and CUA. One of the remaining studies used only CBA, and one used CBA and CUA. The main target population was sexually active people (25 out of 39), including men and women. Most studies (35 out of 39) included women, and 18 studies only focused on women. The total number of participants was ~7,991,198. Twenty-seven out of 39 studies applied major outcomes averted (MOAs), such as PID in women, epididymitis in men, or neonatal pneumonia in newborns. Fifteen studies used QALYs as an outcome measure. Only two studies applied other outcome measures, such as net monetary benefit (NMB) or the number of treated CT cases.

Studies were mostly model-based (38 out of 39). Of 38 studies, 14 applied dynamic models, and 24 used static ones. One study was based on a trial. Twenty-eight studies reported a time horizon for screening or model calculation, ranging from 4 months to individuals' lifetime. Except for 3 studies, other studies conducted a sensitivity analysis to assess the impact of uncertainty assumptions on the results, including univariate or multivariate models with different parameters.

### 3.3. Study findings

Studies were categorized by target populations or settings: general sexually active people (*n* = 25), pregnant women (*n* = 4), women attending STD and abortion clinics (*n* = 4), and other high-risk individuals (*n* = 6).

#### 3.3.1. General sexually active people

Twenty-five studies focused on general sexually active people, nine only included women, two only included men, and 14 included both men and women.

##### 3.3.1.1. Young people in the general population

Sixteen studies focused on young people in the general population (6, 22–25, 28, 29, 33–36, 38, 40–42, 44), six only focused on women, one only focused on men, and 9 focused on both women and men. Despite the heterogeneity of screening strategies in these studies, nearly all studies (13 of 16) yielded similar results. Selective or universal CT screening for young sexually active adults was cost-effective or cost-saving compared with no screening or other interventions at certain conditions. Two studies on men and women in the Netherlands compared different intervals of CT screening. One study found that screening every 2 years was optimal (ICUR <threshold of €20,000 per QALY) (38). Another study found that repeat rounds of CT screening were more cost-effective than no screening only at a high societal willingness to pay > €50,000 per QALY (42). One female-only study from the USA concluded that annual screening for all women aged 15–29 years followed by semiannual screening for those with a history of infection is the most cost-effective strategy (ICUR <threshold of $50,000 per QALY) (29). One male-only study found that the leukocyte esterase (LE)-ligase chain reaction (LCR) test was the most cost-effective strategy in populations with a prevalence of 5% (28).

##### 3.3.1.2. Military recruits and high school students

Three studies focused on school students (27, 37, 43) and 2 studies focused on military recruits, who are young and sexually active and gather in a crowd (21, 39). Two studies performed CT screening programs in several high schools in the USA and found that school-based screening was cost-effective than non-school-based screening if the student participation rate was higher than a certain level (27, 43). A study from Maryland found that screening female military recruits was cost-saving from both military and civilian perspectives (21). Another study from Maryland showed that both selective screening for male military recruits and universal screening for males incorporating female partner notification (PN) were cost-effective (39).

##### 3.3.1.3. Home-obtained sampling method

Three studies from the Netherlands and Sweden were conducted using mailed home-collected urine samples (26, 30, 31). Two studies with asymptomatic young people found that mailing samples was cost-saving when the detection rate was higher than the break-even rate, and the participation rate was relatively high (26, 30). A pilot study found that collaborating with a pharmacy using mailed samples for CT screening was cost-saving for females who collected their contraceptives at the pharmacy (31). Another study comparing home sampling with office sampling (urine specimens for men and vaginal swabs for women) over 10 years concluded that home sampling was cost-saving in the fourth year (32).

#### 3.3.2. Pregnant women

Two studies from Hungary and America concluded that antenatal CT screening for all pregnant women aged 15–24 years was more cost-effective or net cost-saving compared with no screening if the prevalence was higher than a certain level (45, 48). One study from Australia compared antenatal screening in all 16–25-year-old women at their first antenatal visit with no screening or selective screening (aged 16–19 years and/or sexual partners>1 in the last 12 months) and yielded similar results (46). Another study from the Netherlands found that screening all pregnant women was net-cost saving at a test price of up to €20 (47). In addition, the screening was more cost-saving if screening targeted women below 30 years or with first pregnancies.

#### 3.3.3. Women attending STD and abortion clinics

Two studies were conducted on STD clinics (51, 52), one in an abortion clinic (50), and another in multiple high-risk settings, including abortion clinics (49). These studies yielded quite different results as they used different comparators. One study from the UK compared universal screening with clinic-setting-based selective screening and found that selective screening for all women attending abortion clinics was the most cost-effective (ICER was £433 per MOA) (49). A study from China showed that azithromycin-based prophylaxis for women seeking induced abortions was more cost-effective than universal screening at a test prevalence of 4.8% and saved 18239 RMB per PID cases averted (50). A study from the USA conducted in an STD clinic found that the self-obtained vaginal Aptima Combo 2 (AC2) strategy was the most cost-effective, savings $2384 per PID (51). Another Canadian STD clinic-based study found that adding rectal screening both universally and selectively to urogenital screening would be cost-effective if the CA$50,000 <WTP <CA$100,000 (52).

#### 3.3.4. Other high-risk individuals

Four studies from the USA were conducted in high-risk settings (53–56). Two studies were conducted in jails (54, 55), one in detention (53), and one in 4 different settings, including detention and drug treatment center (56). These studies focused on various aspects of CT screening. One study in jail found that universal screening for CT alone was cost-saving when the prevalence of CT was > 7.7% and the prevalence of Neisseria gonorrhea (NG) was <3.7% among females (54). For males, presumptive treatment was cost-saving when the prevalence of CT was <3.7% and the prevalence of NG was <3.8%. Another study in jail showed that screening males with <30 years was more cost-effective than universal screening, with slightly fewer cases identified at half the cost (55). One study in detention found that universal NAAT screening was more cost-effective than selective LE-positive NAAT screening, saving $24,000 (53). Another study in detention and drug treatment center found that screening males could be a cost-effective alternative to screening females when the prevalence of CT was 86% higher in screened males than that in screened females (56).

One study from Australia focused on FSWs and found that mandatory screening of FSWs every 4 weeks for CT was not cost-effective (ICUR> assumed willingness to pay AU$ 5,000 per QALY) (57). One study conducted in HIV treatment centers in the Netherlands focused on MSM and found that adding annual anorectal CT screening to HIV consultation can be cost-saving if these patients seek little (30%) or no (0%) screening elsewhere (58).

#### 3.3.5. Quality assessments

The CHEC scores of the included studies ranged from 11 to 17, indicating moderate (19 studies) and high quality (20 studies) economic evaluation. The last three items had lower fulfillment rates compared to the other 16 items. Only ten studies discussed the generalizability of the results, and five noted ethical aspects of CT screening. The complete quality assessment of each study is presented in Supplementary material.

## 4. Discussion

This systematic review identified 39 economic evaluation reports on CT screening addressing different types of target populations. The studies generally applied a cost-effectiveness or (and) cost-utility analysis mainly focusing on young women. Most of these studies (34) found that CT screening for specific populations was cost-effective or cost-saving in certain conditions.

### 4.1. Young people in the general population

Our findings suggest that CT screening for young women and men is most likely cost-effective or cost-saving.

Young people, especially those aged 15–24, are usually sexually active compared with other age groups (59). Nowadays, aging and infertility are major public health issues, and protecting the fertility of young people has received much attention in many countries. Guidelines from the USA and Canada recommended CT screening for sexually active people younger than 25 years (17, 60). It is well known that serious outcomes associated with CT infection, such as PID, chronic pelvic pain, ectopic pregnancy, and infertility, occur mostly in women. Thus, most studies have focused on women. Men with CT infection often have no apparent symptoms. However, the pathogen can be transmitted from men to women through sexual intercourse. Therefore, CT screening for men also deserves attention. Screening men can also reduce the CT infection rates of women through PN and referral (61). CT infection detection and treatment in men may prevent a large number of adverse outcomes by averting future CT infections in female partners. Therefore, we recommended performing CT screening for young sexually active people aged 15–24 years; however, the age range for screening can be widened or narrowed according to the prevalence of CT infection in different ranges of age.

### 4.2. Military recruits and high school students

Our findings suggest that conducting CT screening in settings with crowds of young and sexually active people (i.e., schools and the army) may be a cost-effective intervention.

In some countries, most adolescents initiate sexual activity during high school or even middle school. A global survey of 38 countries showed that the prevalence of sexual intercourse among adolescents aged 12–15 was 12.3% (62). In the USA, the proportion of sexual intercourse among all students in grades 9–12 varied from 46.8 to 41.2% from 2005 to 2015 (63). Premature sexual intercourse in adolescence can increase the risk of STIs, and Chlamydia trachomatis is the most common pathogen (64). Therefore, we recommended CT screening in high or middle schools, as it can detect and treat asymptomatic CT infections in adolescents who might not seek screening. However, some students might be worried about stigmatization by their peers, resulting in a low participation rate that can affect the cost-effectiveness of CT screening. Hence, conducting school-based mass screening may improve participation rates. Due to the high rates of related sequelae (i.e., PID and CPP) arising from asymptomatic infection in females and the high cost of treatment, many societies and governmental agencies recommend CT screening among females (65, 66).

Although females play an essential role in the military, males comprise the majority of military recruits in many countries. Male recruits could also represent an ideal population for identifying and interrupting the spread of CT infection. Screening males incorporating female PN would be cost-saving if the savings from averted long-term costs of untreated female infection exceed the sum of the costs of male screening and short-term increased female healthcare usage. Therefore, we recommended CT screening for female recruits or male recruits in combination with female PN in armies.

### 4.3. Home-obtained sampling method

Our results suggest that home-sampling CT screening via urine specimens or vaginal swabs (only in women) may be cost-saving in high-prevalence areas. This method can improve the screening participation rate and the number of partners tested (67, 68). A noninvasive first-void urine sample or a self-obtained vaginal swab is usually used. Instructions are provided for collecting and storing the specimens before mailing. This simple approach not only promotes acceptability but also protects individuals' privacy. Therefore, we recommended the home-based sampling method for CT screening. Pharmacies can help CT screening without enough number of STD clinics, as it is more challenging to contact high-risk populations in regions with few STD clinics (31).

### 4.4. Pregnant women

Pregnant women are a specific target group for CT screening. As recommended in several countries, antenatal screening can decrease morbidity among pregnant women and prevent vertical and horizontal transmission. Our study suggests that incorporating CT screening into routine antenatal care for young pregnant women younger than 30 years may be cost-effective or net cost-saving. In addition, treatment for positive patients and PN should be considered to reduce reinfection. However, costs and effects rely on the prevalence of CT, and screening becomes cost-effective or net cost-saving only when prevalence rates are relatively high. Most pregnant women attend an antenatal clinic seeking medical attention for themselves and their fetus, which is a good opportunity to increase the participation rate (90 vs. 37%) (69, 70). Therefore, CT screening can be considered as a part of routine antenatal test based on the prevalence and burden of CT and economic conditions in each region.

### 4.5. Women attending STD and abortion clinics

Opportunistic CT screening for women attending STD and abortion clinics may be cost-effective if strategies and additional costs are acceptable.

Induced abortion can increase the risk of PID and STD. The most common STD is genital chlamydial infection, which can cause asymptomatic salpingitis and subsequent infertility (71–73). In addition, the surgery might spread infection from the lower genital tract to the upper genital tract. We recommended CT screening to women attending abortion clinics to prevent post-abortion complications. A universal prophylactic treatment with azithromycin could be another option. A study in China found that azithromycin-based prophylaxis was cost-saving compared to universal screening (50). The test prevalence (4.8%) of CT in China was lower than that in other developing countries. The study focused on a low-risk population but assumed a post-abortion PID rate of 63%, which may has been overestimated. Thus, more economic evidence should be provided in future studies to approve or disapprove the use of this preventive measure. STD clinics are commonly recognized as high-risk settings but serve women with diverse needs. Some women request only an STD screening and are otherwise healthy. A non-invasive self-obtained vaginal swab not only respects the desires of many women but also saves the resources of the healthcare systems.

Rectal CT screening was proposed by another study from Canada. Extra-genital sites serve as hidden reservoirs for ongoing transmission of infection (74, 75). Rectal CT infection is common in women and is not necessarily associated with anal intercourse. Two epidemiological studies using rectal CT screening for women with a history of anal intercourse found increased rates of CT cases (9.5 and 23%, respectively) (76, 77). However, a similar study reported a 5.8% incidence of rectal-positive cultures in women with no history of anal intercourse (74). Hence, in favorable economic conditions, we recommended a universal or selective (exposure-based) rectal CT screening in combination with urogenital screening for women attending STD clinics. For urogenital screening, an acceptable self-obtained vaginal swab could be widely used.

### 4.6. Other high-risk individuals

CT screening for people entering correctional institutions (i.e., jail, detention) may be a cost-effective intervention. The prevalence of STDs, such as Chlamydial infection and gonorrheal infection, is higher among inmates than in the general population (78). Inmates are often at high risk for STDs but have little access to care. Therefore, the following recommendations should be considered. First, screening programs should test and treat inmates as early after intake as possible to ensure the test results are available before release. Second, it is better to use urine specimen, which is easier to obtain compared to cervical or urethral specimen in jails. Third, the screening should focus on inmates aged 14–30 years; however, the age range can be widened or narrowed based on the prevalence of CT in different age ranges. Fourth, if the prevalence of CT in screened males is well higher than that in screened females, screening high-risk men combining with female PN rather than screening female alone. If the prevalence of gonorrhea was higher than Chlamydia, combined screening for Chlamydia and gonorrhea would be a cost-saving choice.

Sex workers are also at high risk of Chlamydia. One study from Australia suggested that CT screening of FSWs every 4 weeks was not necessary. In Australia, sex work is decriminalized, and sex workers must have regular sexual health check-ups for CT and other STDs. Furthermore, sex workers usually use condoms and have a low incidence of STIs. However, in other countries like China, commercial sex is illegal. A meta-analysis reported that the pooled prevalence of CT infection among FSWs was 16.39% (95% CI: 12.78–20.35%) (79). Moreover, a study conducted in eight cities of 4 provinces in China reported that only 52% of FSWs consistently used a condom during the previous month (80). Therefore, we recommended that CT screening should be provided for FSWs based on the local incidence and prevalence of CT.

Data from many countries showed that MSM have a high burden of HIV and other STDs (81). One study suggested that combing once- or twice-yearly CT screening and HIV care may be a cost-saving program in the Netherlands. Unprotected receptive anal intercourse is a common high-risk sex behavior in MSM. A recent study showed that the positive rate of CT in the rectum, oropharynx, and urethra was 8.0, 0.5, and 3.0%, respectively (82). The highest detection rate of Chlamydia was for rectum. CT infection in an HIV-infected person may increase the transmissibility of HIV, and CT infection in an HIV-uninfected person may increase susceptibility to HIV infection. STD screening is separated from HIV care in some countries like the Netherlands. Many asymptomatic CT-infected MSM may seek screening from elsewhere (i.e., general practitioners, dermatologists, and infectious disease specialists) on the first step, referred to as non-routine screening. However, this non-routine screening is more expensive than adding routine screening. Thus, we recommended integrating the anorectal CT screening of MSM with regular HIV consultation. The proportion of MSM seeking non-routine screening should be limited. As NG and CT can be detected with the same NAAT, future studies can combine NG and CT screening.

### 4.7. Comparison with other studies

We identified only one previous systematic review in this field (18). Roberts et al. reviewed the diagnostic tests, contact tracing, and treatment of CT from a health economic perspective before Aug 2004 (1987–2004). They only included cases with CT and MOAs and did not include QALYs. Furthermore, there was a slight mismatch between the title and the included studies. The title contained only “screening,” but the included studies covered both diagnosis and treatment. Methodological issues seem to persist, partly explained by the lack of enough data for analysis. In summary, our review has increased the outcomes and added QALYs and other indicators. We also updated research progress from 2004 to the present and provided the first narrative by the target population.

## 5. Strengths and weaknesses

This study is the first systematic review assessing CT screening in different populations from a health economic viewpoint. Our search included articles from January 1, 2000, to Match 4, 2023, presenting up-to-date evidence compared to previous reviews.

Two main methodological issues can threaten the validity of these findings. First, most studies used static models inappropriate for the economic evaluation of CT screening. Second, none of the included studies investigated the uncertainty associated with estimates such as the PID progression probability. Moreover, there was a paucity of data on the economic evaluations of CT screening in developing and undeveloped countries. Therefore, countries with different economic conditions need to be cautious when referring to the findings of this review. Overall, our findings indicated that current evidence has limitations, which may impact its interpretation and use in health policy decision-making. Future studies should address these concerns.

## 6. Conclusion

Screening CT among general sexually active people, pregnant women, women attending STD and abortion clinics, people entering correctional institutions, FSWs, and MSM may be cost-effective or cost-saving. CT screening should be considered based on local conditions such as CT prevalence, economic conditions, and cost of screening. A consensus suggests conducting CT screening for general sexually active people and pregnant women in lower age groups. In addition, choosing high-risk settings such as abortion clinics and STD clinics would be a good choice for CT screening. Early screening for new inmates entering correctional institutions is highly recommended. FSWs and MSM deserve attention as they are at high risk of STDs. Given the variations in the results of included studies, we could not draw a firm conclusion for each target population. We could only provide some practical recommendations for developing local screening strategies.

## Data availability statement

The original contributions presented in the study are included in the article/Supplementary material, further inquiries can be directed to the corresponding authors.

## Author contributions

HY and XD participated in the study design and protocol. HY and CL searched the literature, retrieved articles, and drafted the manuscript. HY, CL, and XD screened the articles, extracted the data, and assessed the quality of articles. All authors reviewed the manuscript, figures, and tables.
