# Supplementary material for: Evaluation of Chlamydia trachomatis screening from the perspective of health economics: a systematic review
Source: Front Public Health. 2023 Oct 10;11:1212890. doi: 10.3389/fpubh.2023.1212890 (PMC10595018; doi:10.3389/fpubh.2023.1212890)
Supplement: Supplementary file 1 [file Data_Sheet_1.docx]

Supplementary Material

Health economic evaluation of screening for Chlamydia trachomatis: a systematic review

Huan Yao, Cuizhi Li, Fenglin Tian, Xiaohan Liu, Shangfeng Yang, Qin Xiao, Yuqing Jin, Shujie Huang, Peizhen Zhao, Wenjun Ma, Tao Liu^*^, Xiaomei Dong^*^, Cheng Wang^*^

*** Correspondence:** Cheng Wang:[wangcheng090705@gmail.com](mailto:wangcheng090705@gmail.com); XiaomeiDong:[ntydxm@126.com](mailto:ntydxm@126.com); Tao Liu: [gztt_2002@163.com](mailto:gztt_2002@163.com)

# Search strategy

PubMed

**
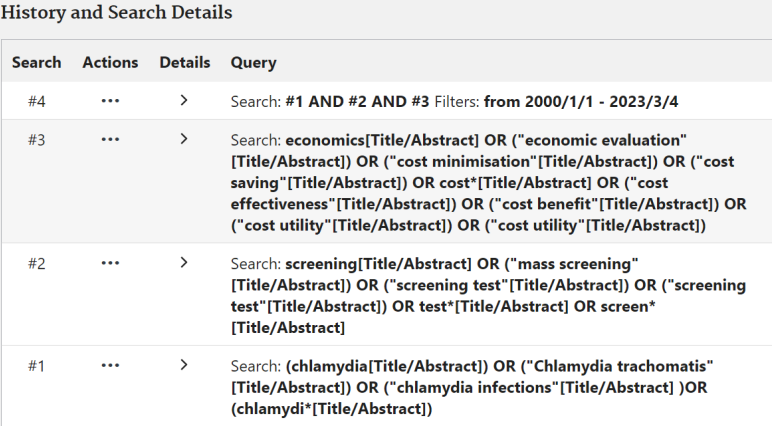
**

Web of science


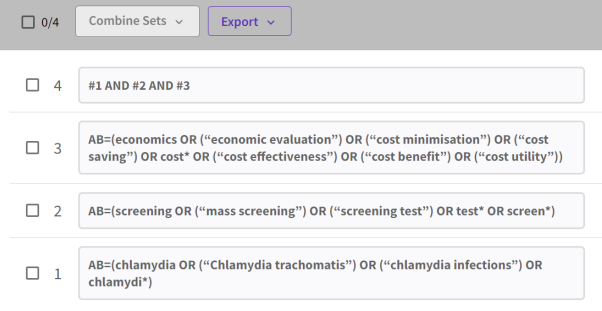


Embase


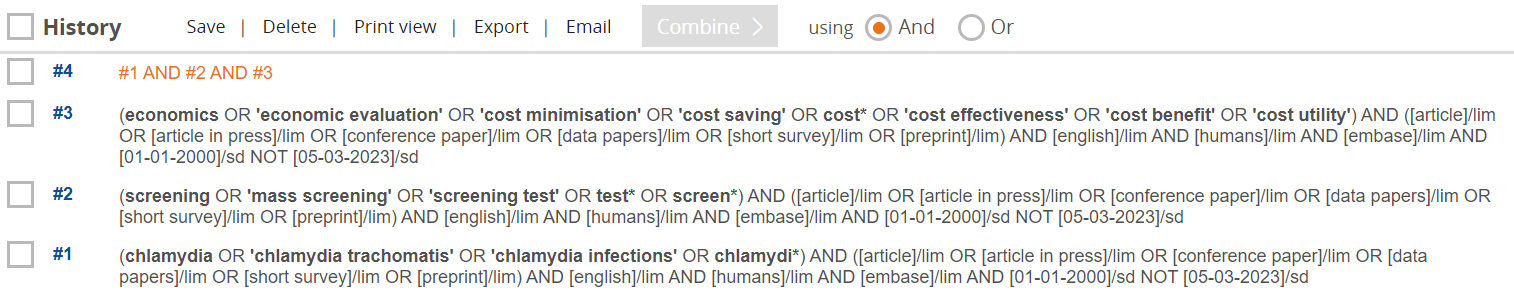


Cochrane library


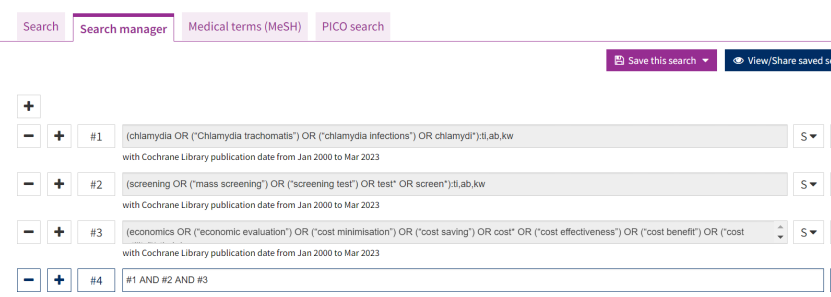


NHS EED via Ovid


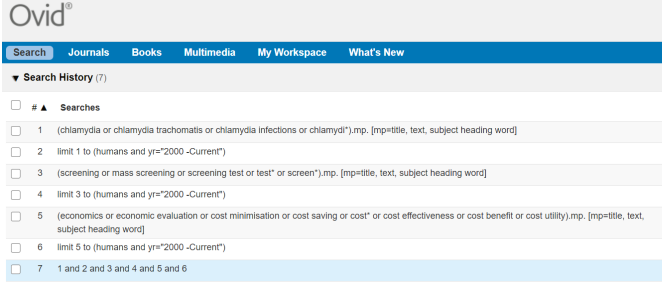


# The CHEC list with the interpreted explanations

| Item number | Explanation |
| --- | --- |
| 1.Is the study population clearly described? | The relevant clinical characteristics, entry and eligibility criteria, as well as drop-out during follow up should be stated explicitly. |
| 2.Are competing alternatives clearly described? | Detailed description of the interventions stated in the article or referenced to another study. |
| 3.Is a well-defined research question posed in answerable form? | Aim of the study should be defined with the alternatives being compared and the population for which the comparison is made. |
| 4.Is the economic study design appropriate to the stated objective? | Appropriate economic evaluation should be used cost-utility or cost-effectiveness analysis. |
| 5. Is the chosen time horizon appropriate in order to include relevant costs and consequences? | Long-term or lifetime time horizon was considered appropriate to include all relevant costs and outcomes. |
| 6.Is the actual perspective chosen appropriate? | Societal perspective was appropriate, but narrower perspective was accepted if the author justified the chosen perspective. |
| 7.Are all important and relevant costs for each alternative identified? | A full identification of all important and relevant costs should be given in relation to the perspective and the research question. |
| 8.Are all costs measured appropriately in physical units? | The costs should be measured appropriately in physical units. The instrument by which the costs are measured should be valid and clearly stated (e.g. interview, questionnaire, cost-diary). |
| 9.Are costs valued appropriately? | The sources of valuation should be clearly stated for each cost price of every volume parameter and their reference year. |
| 10.Are all important and relevant outcomes for each alternative identified? | A full identification of all important and relevant outcomes should be given in relation to the perspective and the research question. |
| 11. Are all outcomes measured appropriately? | The outcome measurement should result from the outcome identification and this should be straightforward. The instrument by which the outcomes are measured should be valid and clearly stated. |
| 12. Are outcomes valued appropriately? | The method of outcome valuation should be clearly stated. |
| 13.Is an incremental analysis of costs and outcomes of alternatives performed? | The box was ticked “yes” is an incremental cost and incremental outcome measures were stated. |
| 14.Are all future costs and outcomes discounted appropriately? | Discounting is done appropriately if all costs and outcomes are converted to one single year, based on a motivated discount rate. Same discount rate should be used for both costs and outcomes. |
| 15.Are all important variables, whose values are uncertain, appropriately subjected to sensitivity analysis? | Sensitivity of all variables should be assessed. A justification should be given over the range of the variables used in the sensitivity analysis. |
| 16.Do the conclusions follow from the data reported? | Do the authors interpret their results cautiously and are their conclusions justified by the data. |
| 17.Does the study discuss the generalizability of the results to other settings and patient/client groups? | This can be done by being explicit about the viewpoint of analysis and by indicating how particular costs and outcomes vary by location, setting, patient population, care provider, etc. |
| 18.Does the article indicate that there is no potential conflict of interest of study researcher(s) and funder(s)? | If an external agency finances the study, a statement should explicitly be given about who finances the study to guarantee transparency in the relationship between the sponsor and the researcher. Whenever a potential conflict of interest is possible a declaration should be given of ‘competing interest’. |
| 19.Are ethical and distributional issues discussed appropriately? | Does the article notes ethical aspects and elaborates on the characteristics of the population experiencing the disease or the intervention (young, old, poor, wealthy) and how this may have distributional implications? |

# Quality assessment of included studies based on the CHEC list

| Author (year of publication) | 1 | 2 | 3 | 4 | 5 | 6 | 7 | 8 | 9 | 10 | 11 | 12 | 13 | 14 | 15 | 16 | 17 | 18 | 19 | Total | Rank |
| --- | --- | --- | --- | --- | --- | --- | --- | --- | --- | --- | --- | --- | --- | --- | --- | --- | --- | --- | --- | --- | --- |
| General sexually active people | | | | | | | | | | | | | | | | | | | | | |
| Howell et al. (2000) | U | U | Y | Y | Y | Y | Y | Y | Y | Y | Y | Y | Y | U | Y | Y | N | N | N | 14.5 | Moderate |
| Postma et al. (2000) | Y | U | Y | Y | N | Y | Y | Y | Y | Y | Y | Y | N | U | Y | Y | N | N | N | 14 | Moderate |
| Welte et al. (2000) | Y | Y | Y | Y | Y | Y | Y | Y | Y | Y | Y | Y | N | Y | Y | Y | Y | N | N | 16 | High |
| Goeree et al. (2001) | U | U | Y | Y | Y | Y | Y | Y | Y | Y | Y | Y | Y | N | Y | Y | N | N | N | 14 | Moderate |
| Nyári et al. (2001) | Y | Y | Y | Y | N | N | Y | Y | N | Y | Y | Y | Y | N | N | Y | N | N | N | 11 | Moderate |
| Van Valkengoed et al. (2001) | U | Y | Y | Y | N | Y | Y | Y | Y | Y | Y | Y | N | U | Y | Y | N | Y | U | 14.5 | Moderate |
| Wang et al. (2002) | U | Y | Y | Y | N | Y | Y | Y | Y | Y | Y | Y | Y | N | Y | Y | Y | N | N | 14.5 | Moderate |
| Ginocchio et al. (2003) | U | U | Y | Y | Y | Y | Y | Y | Y | Y | Y | Y | Y | Y | Y | Y | N | N | N | 15 | High |
| Hu et al. (2004) | U | Y | Y | U | Y | Y | Y | Y | Y | Y | Y | Y | Y | U | Y | Y | Y | Y | N | 16.5 | High |
| Novak et al. (2004) | U | Y | Y | Y | N | Y | Y | Y | Y | Y | Y | Y | Y | N | Y | Y | N | N | U | 14 | Moderate |
| Van Bergen et al. (2004) | U | Y | Y | Y | Y | N | Y | Y | Y | U | Y | Y | N | Y | N | Y | N | N | N | 12 | Moderate |
| Andersen et al. (2006) | U | Y | Y | Y | Y | Y | Y | Y | Y | Y | Y | Y | Y | Y | Y | Y | N | Y | N | 16.5 | High |
| de Vries et al. (2006) | U | U | Y | Y | Y | Y | Y | Y | Y | Y | Y | Y | N | Y | Y | Y | N | N | N | 14 | Moderate |
| Walleser et al. (2006) | U | U | Y | U | Y | Y | Y | Y | U | Y | Y | Y | Y | Y | Y | Y | N | Y | N | 15 | High |
| Adams et al. (2007) | U | Y | Y | U | Y | Y | Y | Y | Y | Y | Y | Y | Y | Y | Y | Y | N | Y | N | 16 | High |
| Roberts et al. (2007) | U | U | Y | Y | Y | Y | Y | Y | Y | Y | Y | Y | Y | Y | Y | Y | N | Y | N | 16 | High |
| Blake et al. (2008) | Y | U | Y | Y | Y | Y | Y | Y | Y | Y | Y | Y | Y | U | Y | Y | Y | N | N | 16 | High |
| De Vries et al. (2008) | U | Y | Y | U | Y | Y | Y | Y | Y | Y | Y | Y | Y | Y | N | Y | N | N | N | 14 | Moderate |
| Nevin et al. (2008) | U | U | Y | Y | Y | Y | Y | Y | Y | Y | Y | Y | Y | U | Y | N | N | N | N | 13.5 | Moderate |
| Huang et al. (2011) | U | U | Y | Y | Y | Y | Y | Y | Y | Y | Y | Y | Y | Y | Y | Y | Y | Y | N | 17 | High |
| Gillespie et al. (2012) | U | Y | Y | U | Y | Y | Y | Y | Y | Y | Y | Y | Y | Y | Y | Y | N | Y | U | 16.5 | High |
| Tuite et al. (2012) | U | Y | Y | U | Y | Y | Y | Y | Y | Y | Y | Y | Y | Y | Y | Y | N | N | N | 15 | High |
| de Wit et al. (2015) | Y | Y | Y | U | Y | Y | Y | Y | U | Y | Y | Y | Y | Y | Y | Y | N | Y | N | 16.5 | High |
| Wang et al. (2021) | U | Y | Y | U | Y | Y | Y | Y | Y | Y | Y | Y | N | Y | Y | Y | N | Y | N | 15 | High |
| Stoecker et al. (2022) | U | U | Y | U | Y | Y | Y | Y | Y | Y | Y | Y | N | U | Y | Y | Y | Y | N | 15 | High |
| Pregnant women | | | | | | | | | | | | | | | | | | | | | |
| Nyári et al. (2003) | Y | U | U | Y | Y | N | Y | Y | U | Y | Y | Y | Y | N | Y | Y | N | N | N | 12.5 | Moderate |
| Ong et al. (2015) | U | Y | Y | U | Y | Y | Y | Y | Y | Y | Y | Y | Y | N | Y | Y | Y | Y | N | 16 | High |
| Rours et al. (2016) | U | U | Y | U | N | Y | Y | Y | U | Y | Y | Y | N | Y | Y | Y | Y | Y | N | 14 | Moderate |
| Ditkowsky et al. (2017) | U | U | Y | Y | Y | Y | Y | Y | Y | Y | Y | Y | N | N | Y | Y | N | Y | U | 14.5 | Moderate |
| Women attending clinics | | | | | | | | | | | | | | | | | | | | | |
| Norman et al. (2004) | Y | Y | Y | Y | N | Y | Y | Y | Y | Y | Y | Y | Y | Y | Y | Y | N | N | U | 15.5 | High |
| Chen et al. (2007) | U | U | Y | Y | U | Y | Y | Y | Y | Y | Y | Y | Y | U | Y | Y | N | N | N | 14 | Moderate |
| Blake et al. (2008) | U | U | Y | Y | Y | Y | Y | Y | Y | Y | Y | Y | Y | U | Y | Y | N | N | N | 14.5 | Moderate |
| Thanh et al. (2017) | U | U | Y | U | Y | Y | Y | Y | Y | Y | Y | Y | Y | Y | Y | Y | N | Y | N | 15.5 | High |
| Other high-risk individuals | | | | | | | | | | | | | | | | | | | | | |
| Blake et al. (2004) | U | Y | Y | Y | Y | Y | Y | Y | Y | Y | Y | Y | Y | U | Y | Y | Y | N | N | 16 | High |
| Kraut-Becher et al. (2004) | U | U | Y | Y | N | Y | Y | Y | Y | Y | Y | Y | Y | U | Y | Y | N | N | N | 13.5 | Moderate |
| Gift et al. (2006) | U | Y | Y | Y | Y | Y | Y | Y | Y | Y | Y | Y | Y | U | Y | Y | N | N | N | 15 | High |
| Gift et al. (2008) | U | Y | Y | U | Y | Y | Y | Y | Y | Y | Y | Y | Y | Y | Y | Y | N | N | N | 15 | High |
| Wilson et al. (2010) | U | U | Y | U | N | Y | Y | Y | U | Y | Y | Y | N | Y | N | Y | Y | Y | N | 13 | Moderate |
| Vriend et al. (2013) | U | Y | Y | U | Y | N | Y | Y | U | Y | Y | Y | Y | Y | Y | Y | N | Y | N | 14.5 | Moderate |
